# Atraumatic surgical technique for post‐traumatic ingrown fingernails

**DOI:** 10.1111/ddg.15698

**Published:** 2025-04-10

**Authors:** Simona Sabulyte, Galina Balakirski, Christoph R. Löser

**Affiliations:** ^1^ Zentrum für Dermatologie Allergologie und Dermatochirurgie Universitätsklinikum Wuppertal Universität Witten/Herdecke, Wuppertal; ^2^ Hautklinik Hauttumorzentrum Klinikum der Stadt Ludwigshafen gGmbH Ludwigshafen am Rhein

**Keywords:** ingrown nail, nail surgery, nail trauma, post‐traumatic nail changes, Unguis incarnatus

## INTRODUCTION

The fingernail has great aesthetic significance and is functionally imperiled by crude surgical manipulations. The use of traumatic surgical methods regularly results in irreversible damage with functional and/or aesthetic impairment.^1^ Compared to ingrown toenails, the condition of ingrown fingernails is hardly mentioned in the literature. We therefore present two clinical cases on gentle techniques for the surgical treatment of post‐traumatic ingrown fingernails.

## TECHNIQUE

### Case 1

A 44‐year‐old craftsman reported pain in his left middle finger, which affected him both at work and in his daily life. An enchondroma of the distal phalanx, which was diagnosed after multiple spontaneous fractures, was treated about 8 years ago by enchondroma excision and filling of the bone defect by autogenous spongiosa transplantation from the radius. Following enchondroma recurrence, the procedure was repeated two years ago with access to the bone through the nail bed. The nail plate was initially damaged. Just a few months after the procedure, the patient complained increasingly about the ingrowing nail.

**FIGURE 1:**
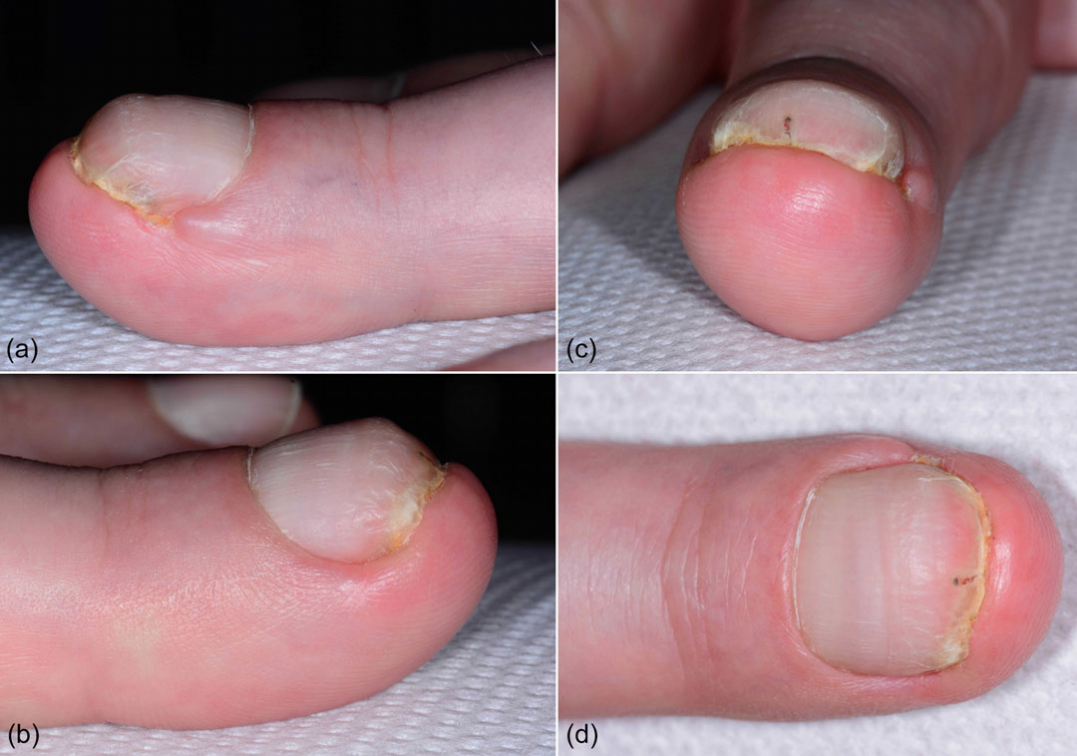
Preoperative findings with pincer nail and distal ingrowth of the nail on the left middle finger. (a, b) The lateral view shows clear lateral ingrowth of the nail plate. (c, d)

**FIGURE 2:**
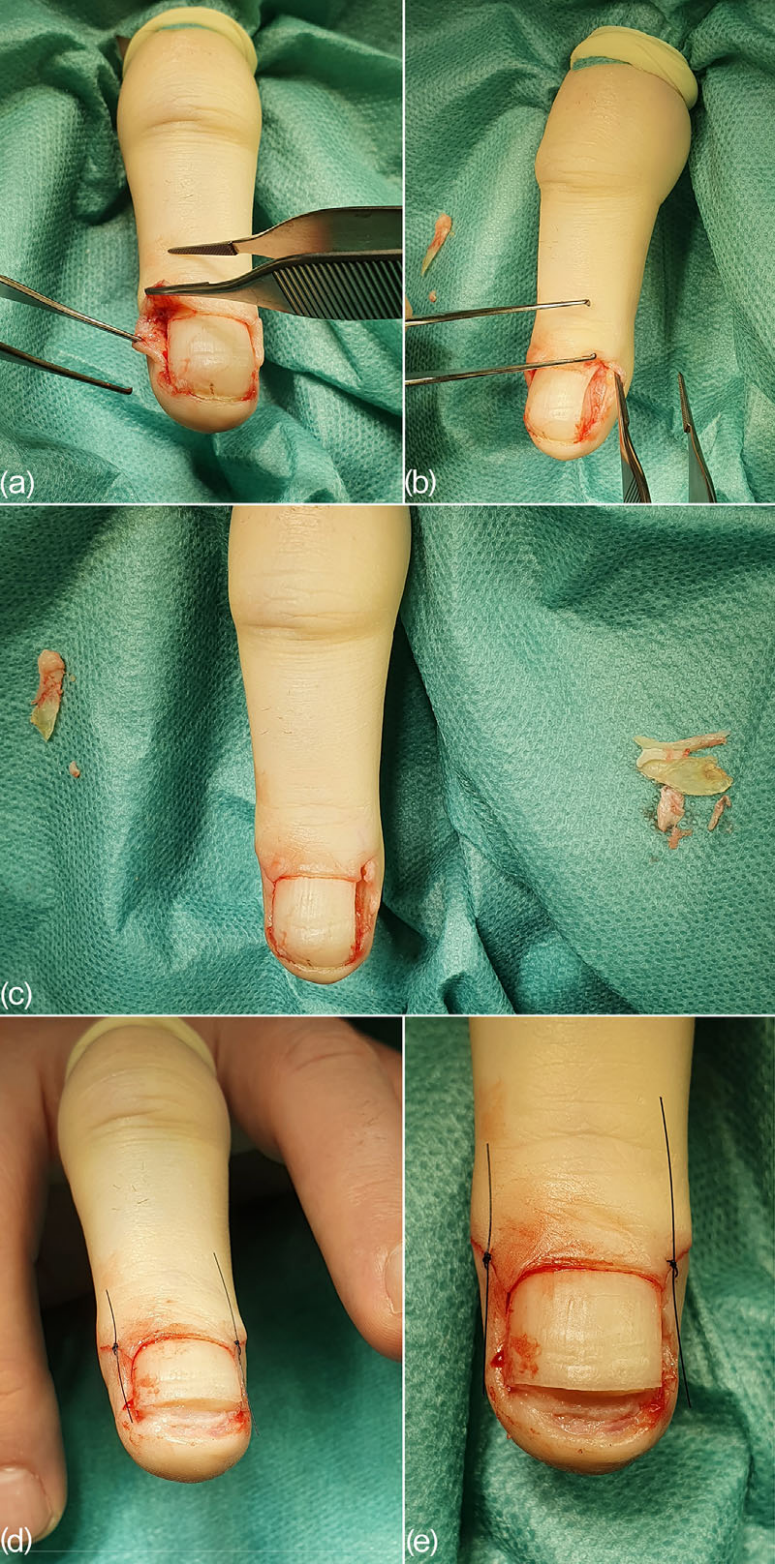
(a–c) Intraoperative view of partial resection of the lateral nail plates and lateral matrix horns and (d, e) distal resection of the nail plate and superficial parts of the nail bed. It should be noted that matrix horn resections are not shown here.

Clinically, there was a clear pincer nail with lateral and distal ingrown areas (Figure 1a–d). Under the digital block anesthesia according to Oberst, a partial resection of the laterally ingrown nail plate and removal of the lateral matrix horns (Figure 2a–c) as well as a distal resection of the downward curved nail plate with superficial sections of the nail bed (Figure 2d–e) were performed.

**FIGURE 3:**
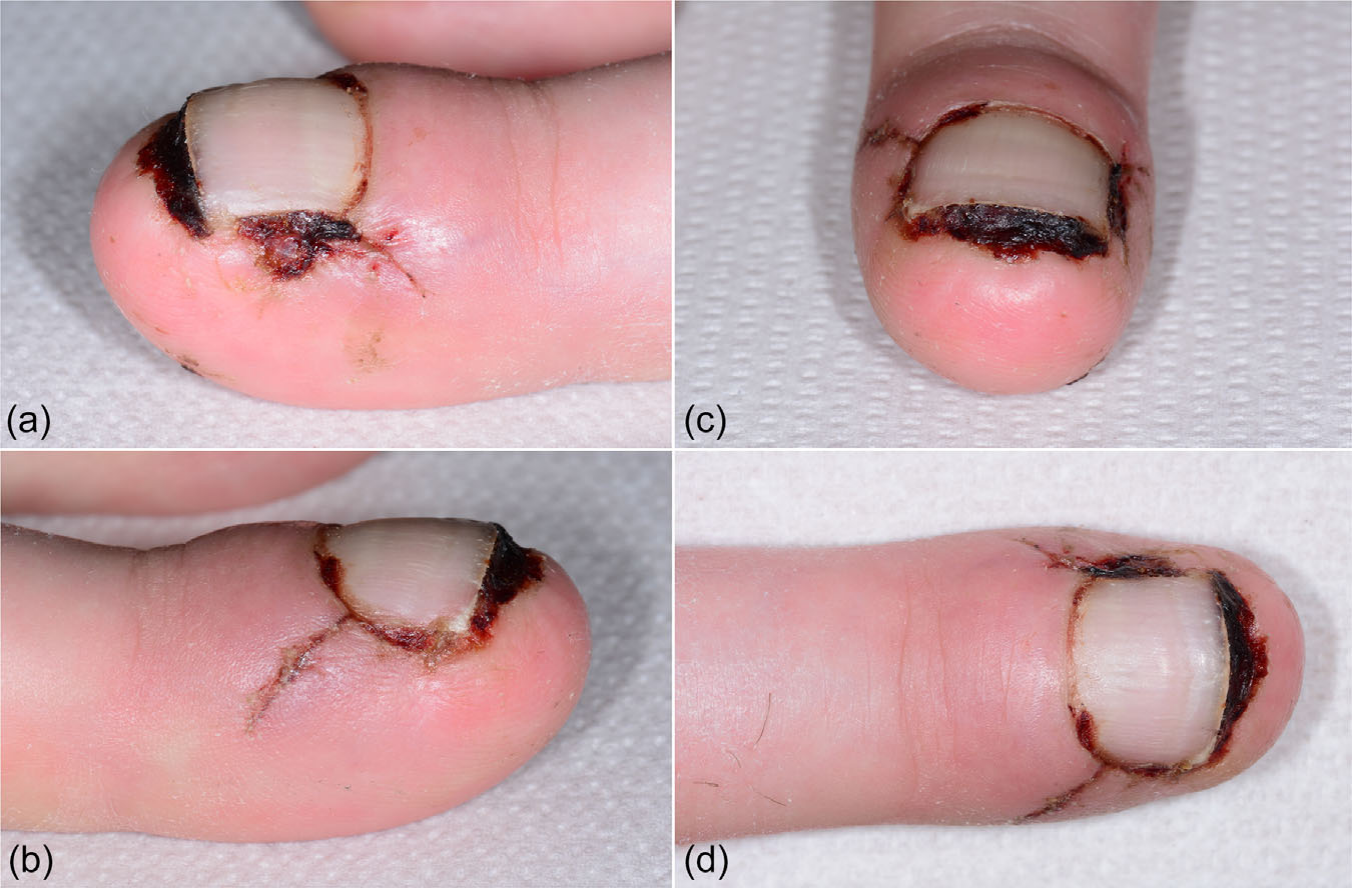
Clinical course of suture removal approximately 2 weeks postoperatively.

**FIGURE 4:**
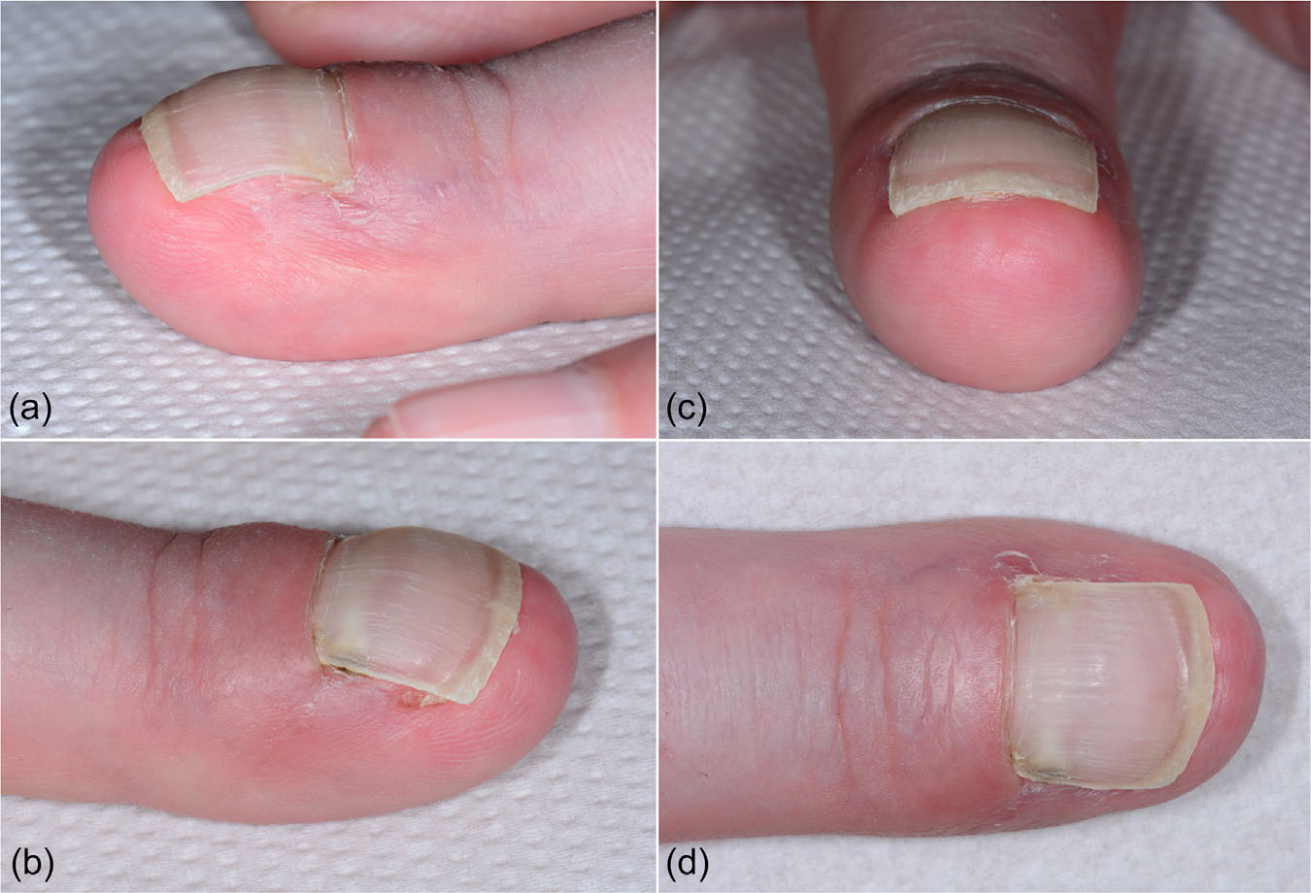
Clinical findings 8 weeks postoperatively. The nail completely covers the nail bed with no evidence of the ingrown areas into the distal nail wall.

Upon suture removal, about 2 weeks postoperatively (Figure 3a–d), and at the follow‐up examination 8 weeks after the procedure, a normal nail regrowth was observed (Figure 4a–d). After healing, the patient was once again completely pain‐free.

### Case 2

**FIGURE 5:**
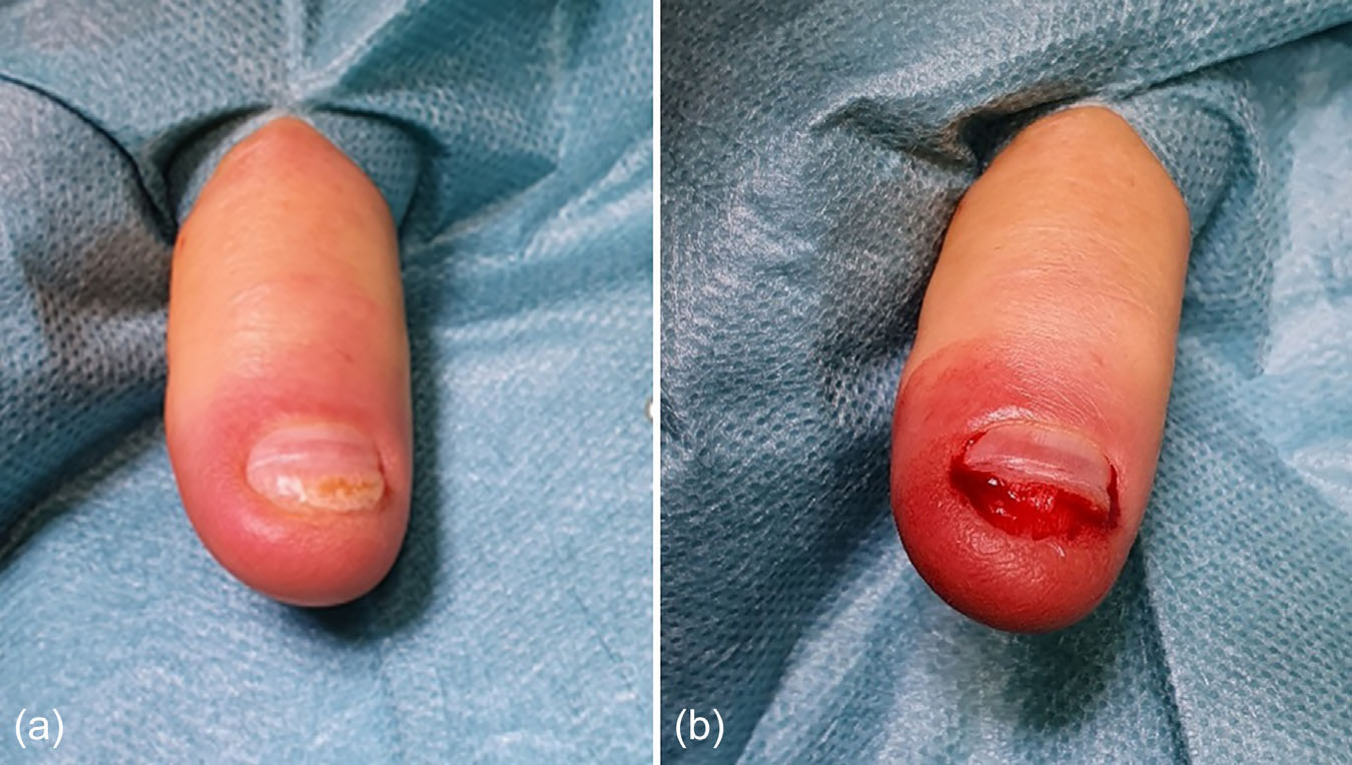
(a) Preoperative findings with painful distal ingrowth of the nail plate with incipient retronychia and (b) surgical correction by shortening the nail plate with removal of the ingrown nail section and surgical debridement of the nail bed.

**FIGURE 6:**
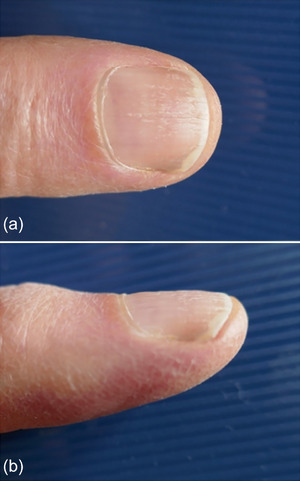
(a, b) Follow‐up with complete regrowth of the nail plate over the nail bed a few months postoperatively (images of the patient, with kind permission).

A 74‐year‐old patient suffered a contusion injury caused by a tailgate, which resulted in the loss of the nail plate on his right thumb. During regrowth of the nail plate, a very painful distal ingrowth of the nail plate occurred, with developing retronychia (Figure 5a). The ingrown nail was removed under the digital block anesthesia accorting to Oberst and the curettage of the nail bed was performed (Figure 5b). The patient was advised to follow up treatment with rehydrating care of the nail bed and massages along the direction of nail growth. At the follow‐up examination a few months after the procedure, the nail had grown back properly and the patient was completely free of pain (Figure 6a,b).

## DISCUSSION

Ingrown toenails are a common condition that is well known among dermatologists.^2^, ^3^ In contrast to this, ingrown fingernails are a relatively rare and less known condition that is barely described in the literature.^4^ Ingrown fingernails are observed particularly in chronic paronychia under therapy with epidermal growth factor receptor (EGFR) inhibitors or as a post‐traumatic state.^4^

A classification into subtypes has been proposed for ingrown toenails^5^ and can also be used for fingernails, since it serves only to describe the site of the problem: Distal embedding, subcutaneous ingrown nail in the case of a nail plate that is too wide, hypertrophy of the lateral nail fold and pincer nail.^5^ Especially following a previous trauma with partial or complete loss of the nail, the regrowing nail plate may become excessively curved longitudinally, causing the free edge of the nail plate to grow painfully into the distal nail wall.^6^ In such cases, partial resection of the nail plate and superficial parts of the nail bed can lead to an improvement in the condition. The longitudinal hypercurvature of the nail bed resulting from the distal ingrowth of the nail plate can be approximated to the original state, parallel to the distal phalanx. In most cases, further growth will uniformly restore the nail to its original shape over the nail bed.^7^ An important recommendation after surgical correction of the distally ingrown nail is to protect exposed nail bed areas from drying out with daily use of suitable topical agents (e.g. Vaseline) and, if necessary, to massage the fingertip away from the finger to counteract retraction and thus a renewed shortening of the nail bed.^8^, ^9^ Conservative treatment approaches involving massage and taping of the distal nail wall have also been described. However, success is usually only visible after several months,^8^ and the duration of remission after conservative treatment has ended is unclear. The authors therefore recommend combining both approaches, atraumatic surgical methods and conservative follow‐up treatment, to achieve lasting improvement of the symptoms.

In summary, minimally invasive correction options are available also for painful ingrown fingernails. As demonstrated in our cases, these options help to avoid extensive corrections of the nail bed (e.g. Howard‐Dubois technique) or now obsolete procedures (such as unspecific nail extraction).

## FUNDING

Dr. Simona Sabulyte received a 2024 fellowship grant for the support of dermatosurgery from the German Dermatological Society (DDG) and the German Society for Dermatosurgery (DGDC).

## CONFLICT OF INTEREST STATEMENT

None.
